# EEG correlates of anxiety in young female depressive patients

**DOI:** 10.1192/j.eurpsy.2026.11416

**Published:** 2026-07-31

**Authors:** A. F. Iznak, E. V. Iznak, E. V. Damyanovich, A. F. Beresneva, I. V. Oleichik

**Affiliations:** 1 Laboratory of Neurophysiology; 2 Department of endogenous mental disorders and affective conditions, Mental Health Research Centre, Moscow, Russian Federation

## Abstract

**Introduction:**

Anxiety is one of the main components of depressive disorder. Comorbidity of depression and anxiety reaches 90%, which increases the overall severity of the clinical condition of depressive patients. Symptoms of anxiety often precede the manifestation of depression, and, according to one of the concepts, anxiety caused by chronic stress can be the cause of the development of depression. The features of the reflection of the severity of anxiety in the EEG patterns of depressive patients have not been sufficiently studied.

**Objectives:**

The aim of the study was clarifying the EEG correlates and neurophysiological mechanisms of anxiety in depressive patients.

**Methods:**

40 female in-patients aged 16-25 years old (mean age 19.4 ± 3.9 years) with depressive disorders (F31.3-4 and F34.0 by ICD-10) were included in the study. Using the method of hierarchical cluster analysis, they were divided into two groups (by 20 patients each) based on the sum of points of the anxiety cluster of the HDRS-17 scale (sum of items 9, 10 and 11) and on the index of the anxiety scale of the SCL-90-R questionnaire. Groups did not differ statistically neither in age, nor in the total score of the depression cluster of the HDRS-17 scale (sum of items 1, 2, 3, 7 and 8) and in the index of the “depression” scale of the SCL-90-R. Resting-state EEG recordings with consequent analysis of absolute EEG spectral power in 8 narrow frequency sub-bands were conducted before the start of treatment. The statistics included the Mann-Whitney test and Spearman correlation analysis.

**Results:**

The group of patients with a greater anxiety had greater overall severity of the clinical and psychological states due to anxiety spectrum disorders (anxiety, phobia, sleep disorders, somatization). The EEG of patients in the group with high anxiety significantly (p<0.05) differed from the EEG of patients with low anxiety by lower values of alpha-2 (9-11 Hz) spectral power in both occipital leads (O1 and O2) and by higher values of alpha-3 (11-13 Hz) spectral power in the anterior temporal (F7, F8) and middle temporal (T3, T4) leads of both hemispheres, as well as in the left central lead (C3). Such spatial-frequency pattern represented the EEG signs of inhibition deficit in the frontotemporal and occipital regions of the cerebral cortex in the group with high anxiety.

**Conclusions:**

The data obtained emphasize the important role of inhibition deficit in the manifestation of anxiety spectrum disorders in the clinical pattern of depression and can be used in the future for clarifying the individual prognosis and optimization of the depression treatment.

**Disclosure of Interest:**

None Declared

